# Regional variation in suicide rates in Sri Lanka between 1955 and 2011: a spatial and temporal analysis

**DOI:** 10.1186/s12889-016-3961-5

**Published:** 2017-02-14

**Authors:** Duleeka W. Knipe, Prianka Padmanathan, Lal Muthuwatta, Chris Metcalfe, David Gunnell

**Affiliations:** 1 South Asian Clinical Toxicology Research Collaboration (SACTRC), Faculty of Medicine, University of Peradeniya, Peradeniya, Sri Lanka; 2 School of Social and Community Medicine, University of Bristol, Canynge Hall, 39 Whatley Road, Bristol, BS8 2PS UK; 3 International Water Management Institute, 127, Sunil Mawatha, Pelawatt, Battaramulla, Sri Lanka

**Keywords:** Suicide, Pesticides, Sri Lanka, Spatial, Temporal, Regional, Socioeconomic, Poisoning, Epidemiology

## Abstract

**Background:**

Between 1955 and 2011 there were marked fluctuations in suicide rates in Sri Lanka; incidence increased six-fold between 1955 and the 1980s, and halved in the early 21st century. Changes in access to highly toxic pesticides are thought to have influenced this pattern. This study investigates variation in suicide rates across Sri Lanka’s 25 districts between 1955 and 2011. We hypothesised that changes in the incidence of suicide would be most marked in rural areas due to the variation in availability of highly toxic pesticides in these locations during this time period.

**Methods:**

We mapped district-level suicide rates in 1955, 1972, 1980 and 2011. These periods preceded, included and postdated the rapid rise in Sri Lanka’s suicide rates. We investigated the associations between district-level variations in suicide rates and census-derived measures of rurality (population density), unemployment, migration and ethnicity using Spearman’s rank correlation and negative binomial models.

**Results:**

The rise and fall in suicide rates was concentrated in more rural areas. In 1980, when suicide rates were at their highest, population density was inversely associated with area variation in suicide rates (*r* = −0.65; *p* < 0.001), i.e. incidence was highest in rural areas. In contrast the association was weakest in 1950, prior to the rise in pesticide suicides (*r* = −0.10; *p* = 0.697). There was no strong evidence that levels of migration or ethnicity were associated with area variations in suicide rates. The relative rates of suicide in the most rural compared to the most urban districts before (1955), during (1980) and after (2011) the rise in highly toxic pesticide availability were 1.1 (95% CI 0.5 to 2.4), 3.7 (2.0 to 6.9) and 2.1 (1.6 to 2.7) respectively.

**Conclusions:**

The findings provide some support for the hypothesis that changes in access to pesticides contributed to the marked fluctuations in Sri Lanka’s suicide rate, but the impact of other factors cannot be ruled out.

## Background

Suicide is a significant cause of mortality worldwide resulting in approximately 800,000 deaths per year [1]. Low- and middle-income countries in the WHO's South-East Asian region account for 39.1% of suicides around the world despite only making up 25.9% of the population [1]. Globally, at least one third of suicides are attributable to pesticide self-poisoning; this proportion is higher in many parts of Asia [2].

Case fatality from pesticide self-poisoning is approximately 10–20% [3]; this is over ten times higher than following self-poisoning in industrialised countries, where medicines are the most commonly ingested poisons [4]. Despite this, many acts of self-poisoning with pesticides are carried out with low suicidal intent [5, 6]. The high case-fatality associated with pesticide self-poisoning combined with the observation that a large proportion of cases have low intent, underpins the importance of pesticides as a major public health issue [7].

Sri Lanka, a middle-income country in South Asia where pesticides account for a high proportion of suicides, has experienced marked fluctuations in its suicide rate over the last 50 years. The highest suicide rate (47 per 100,000) was observed in 1995 [8]. During some of this time period Sri Lanka was involved in a civil war. Analyses however suggest that the fluctuations in suicide rates were driven by changes to the availability of pesticides within the country rather than the conflict [8, 9]. The main changes to the availability of pesticides in Sri Lanka were the result of regulatory activity by the Registrar of Pesticides [10]. More recently the Presidential Committee’s National Suicide Prevention Strategy (1997) included a focus on reducing pesticide accessibility through research, education and legislation.

Research to date has however only investigated fluctuations in suicide rates at a national level. Previous work suggests that internal migration within Sri Lanka may have contributed to the rise and regional differences in suicide rates [11]. Furthermore international literature highlights unemployment [12] and low socioeconomic position [13] as other contributors to suicide trends and area differences in rates.

We hypothesise that if ease of access to pesticides was the main driver for the high suicide rate, the rise and fall in suicide rates would be greatest in agricultural (rural/low population density) areas because of the high levels of pesticide use (ease of access) in these locations. In addition the largest area differences in suicide rates would occur in the 1980s, around the time when high toxicity pesticides were most readily available and self-poisoning accounted for almost 80% of suicides in Sri Lanka [9].

## Methods

### Context

Sri Lanka is an island nation situated in the Indian Ocean, off the South-East coast of India. It has a population of 20.3 million people, 77% of whom live in rural areas [14]. Following a number of boundary changes over the last few decades, the country is composed of 25 districts.

The Sri Lankan civil war (1983–2009) largely took place in the northern and eastern provinces, which include the districts of Jaffna, Mannar, Kilinochchi, Vavuniya, Mullativu, Trincomalee, Batticaloa and Ampara. The capital city of Colombo is situated on the west coast of Sri Lanka. It has the highest population density in the country, followed by its neighboring districts. Agriculture forms the second largest industry in Sri Lanka, employing 28.5% of the working population in 2014 [15].

### Population data

**Fig. 1:**
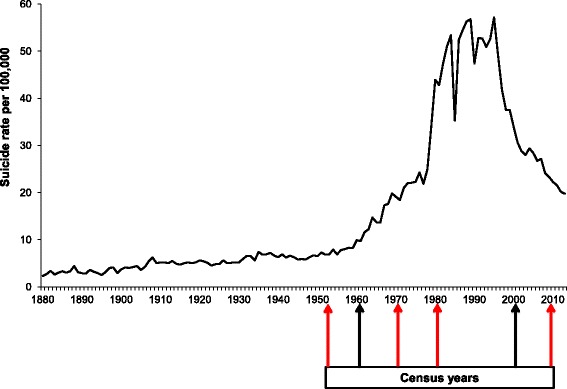
Graph showing national suicide rates over time, and census years. Years selected for analysis in this study are highlighted in *red*

Population data were obtained using the Sri Lankan censuses carried out in 1953, 1971, 1981 and 2011 [14, 16–18]. The first year for our analysis, 1953, was selected as it preceded the year on year rises in suicide that occurred over the subsequent 40 years (Fig. 1) [8]. Data from two censuses (1961 and 2001) were excluded from our analyses because district-level data on suicide risk factors or suicide (see below) were not available for all districts; the 1963 census did not include data broken down by district-level, and the 2001 census omitted data from a number of north-eastern districts.

### Suicide data

District-level data on the incidence of suicide by pesticide poisoning was not available, so all analyses were based on overall suicide rates. Data for 2011 were obtained from the Department of Police, Division of Statistics, Sri Lanka. The number of suicides was initially reported according to 43 police divisions. Suicide rates for each of the 25 districts have been calculated by dividing the total number of suicides for the police divisions that make up each district, by the district population according to the 2011 census (see Appendix 1). Some districts included more than one police division. Where this was the case the division’s boundaries fell entirely within the district; no police division straddled district boundaries. District-level suicide data collected by the Police department for 1955, 1972 and 1980 were obtained from publications by Kearney and Miller (1985, 1988) [6, 7]. For 1955, 1972 and 1980, data were only available on suicide rates; it was not possible to obtain the suicide counts for each district from the Sri Lankan Police Department. We translated these rates into counts by multiplying the district population recorded in the census for each year by the suicide rate for that district.

### Potential risk factors

Potential area-level risk factors for suicide were included in the analysis if: a) there was previous evidence or speculation regarding their association with suicide rates and b) comparable district-level data were available in at least three of the four censuses included in our analysis. This limited the factors available for inclusion.

Where available, data on the following factors were extracted for each district from censuses in 1953 [16], 1971 [17], 1981 [18] and 2011 [14]: a) population density in persons per square mile: a commonly used measure of rurality and hence farming and access to/use of pesticides [19, 20]; b) migration as indexed by the percentage of in-migrants to each district; c) unemployment in terms of the percentage of people over 10 years old in 1971 and 1981, and over 15 years in 2011, who were unemployed but available for work; d) ethnicity with regards to the percentage of Tamils living in each district. In Sri Lanka the main ethnic group, Sinhala, make up 74.9% of the population, whilst Tamils make up 15.3% [9]. Given Sri Lanka’s recent history of civil war involving the Tamil population mainly situated in the northern and eastern provinces, we were interested in investigating whether any changes in the geographical distribution of suicide were associated with the geographical distribution of the Tamil population. Differences in suicide risk between ethnicities have been hypothesised in Sri Lanka but have not been studied [21].

District-level data on religion were also available in each of the four censuses; the main religion in Sri Lanka is Buddhism, accounting for 70% of the population [9]. We note however that the percentage of Buddhists in each district was strongly inversely correlated with the percentage of Tamils at every time point, as Buddhists tend to be Sinhala (all *r* > −0.66, all *p* < 0.01). We have therefore not investigated religion separately.

### Analyses

District-level relative rates of suicide, using the overall national suicide rate as the denominator, were calculated for each time point. These were transposed onto thematic maps created using ArcGIS. We chose standard cut-offs that have been used in previous literature: <0.67, 0.67–0.90, 0.91–1.10, 1.11–1.50, >1.50 [22]. We used the administrative boundary layer package in order to create these maps [23].

The following administrative boundaries for Sri Lanka have changed over the time period investigated, with the formation of:

1. Ampara in 1961 out of the southern part of Batticaloa
2. Gampaha in 1978 out of the northern part of the Colombo district
3. Mullativu in 1978 out of part of the Jaffna district
4. Kilinochchi in 1984 out of the southern part of the Jaffna district

We used the most recent administrative boundaries to create thematic maps for each time point and assumed that for the earlier time points, suicide rates in newly formed districts were the same as for districts from which they were formed. For example for the 1953, 1971 and 1981 data we have assumed that Kilinochchi had the same suicide rate as Jaffna, the district which it was part of until 1984. We compared the rates of newly formed districts with the original district definition in order to check our assumption (see Appendix 2). The differences in suicide rates between newly formed and original districts were slight (<15%) for all districts except Ampara and Batticaloa (40%).

The ecological associations between socioeconomic indicators and suicide rates at each point in time were investigated using Spearman’s rank correlation coefficients. Suicide rates were then stratified by population density to explore the relative risk of suicide according to this proxy indicator of pesticide availability. The following categories were chosen >2000, 500–2000 and <500 persons per square mile. Colombo and Gampaha (combined population in 1981: 3,090,103) were the only two districts with a population density greater than 2000 persons per square mile. Eleven districts (population: 8,105,251) had a population density between 500 and 2000 persons per square mile. Twelve districts (population: 3,651,396) had a population density of <500 persons per square mile. The first cut point was chosen due to the large difference between population densities of Gampaha and Kandy (the third most densely populated district). The second cut point was based on the distinction between dry and wet zones, as this is commonly used to differentiate between intermediate and rural districts in Sri Lanka [24]. We categorised each district at each time point into the 3 population density categories based on the 1981 population density as there was a very strong correlation between the population density in each census year compared with the population density in 1981 (all *r* > 0.98; all *p* < 0.001), indicating that there were no major changes in the relative rurality of the different areas over the course of our analysis.

We estimated the association between the area level suicide rate and population density (as a categorical covariate) as rate ratios, whilst controlling for any confounding by area level unemployment (as a continuous covariate) using negative binomial regression models. Negative binomial regression is an extension of log-linear or Poisson regression (i.e. log transformed rates are included in the model), which accommodates variation between areas in the rate of suicide (using a gamma distributed random effect).

## Results

**Fig. 2:**
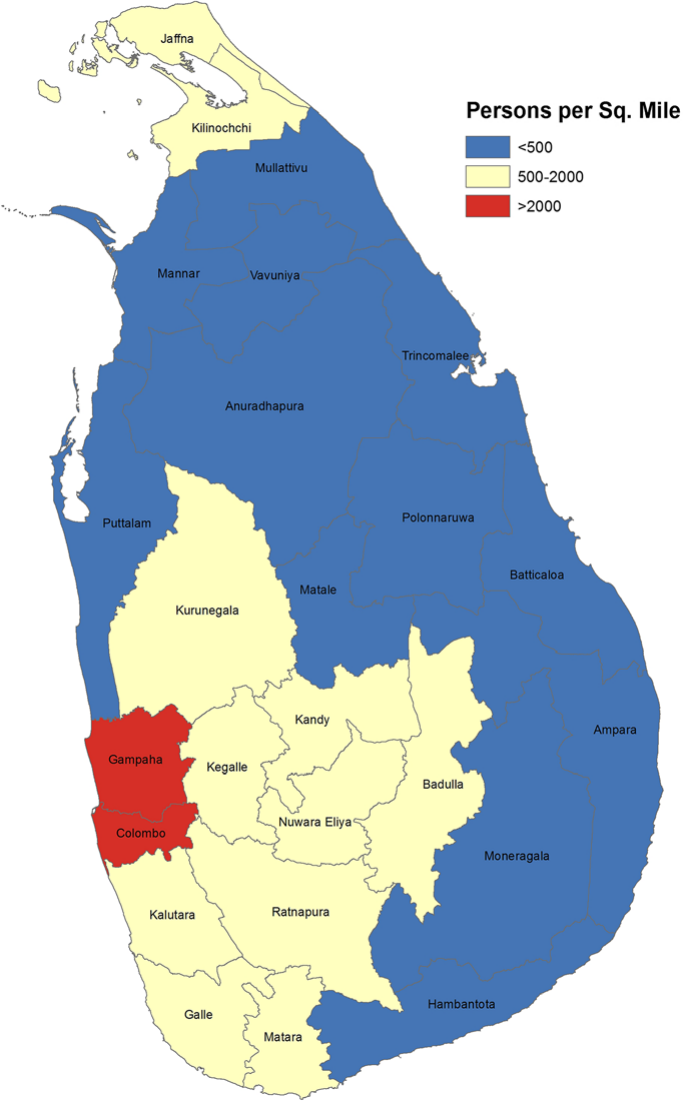
Map of Sri Lanka showing the population densities in each district in 1981

Figure 2 illustrates the distribution of Sri Lanka’s population by district in 1981, using population density as an indicator of rurality or agricultural activity.

There were four fold differences in overall suicide rates in Sri Lanka across the four periods forming the basis of this analysis. The incidence of suicide ranged from 6.9 per 100,000 in 1955 to 29.0 per 100,000 in 1980 (Table 1); male rates were two to three times higher than the female rates. Population density more than doubled between 1955 and 2011; unemployment and the proportion of Tamils in the population fell substantially over the study period.

**Table 1:** Crude suicide rates and socioeconomic factors at each time point

	Crude suicide rate per 100,000				Socioeconomic Factors			
Years	Overall	Male	Female	Census years	Population density^a^	Migration^b^	Unemployment^c^	% Tamil
1955	6.9	9.1	4.5	1953	320	-	-	23.0
1972	21.0	30.1	11.1	1971	509	15.0	18.7	20.5
1980	29.0	37.7	19.7	1981	609	10.7	17.9	18.2
2011	18.5	30.0	7.9	2011	790	19.4	2.6	15.3

^a^Population/square mile, ^b^% in-migrants, ^c^% aged 10 (1971 & 1981) or 15 (2011) and over and available to work

### Overall suicide rates by district

**Fig. 3:**
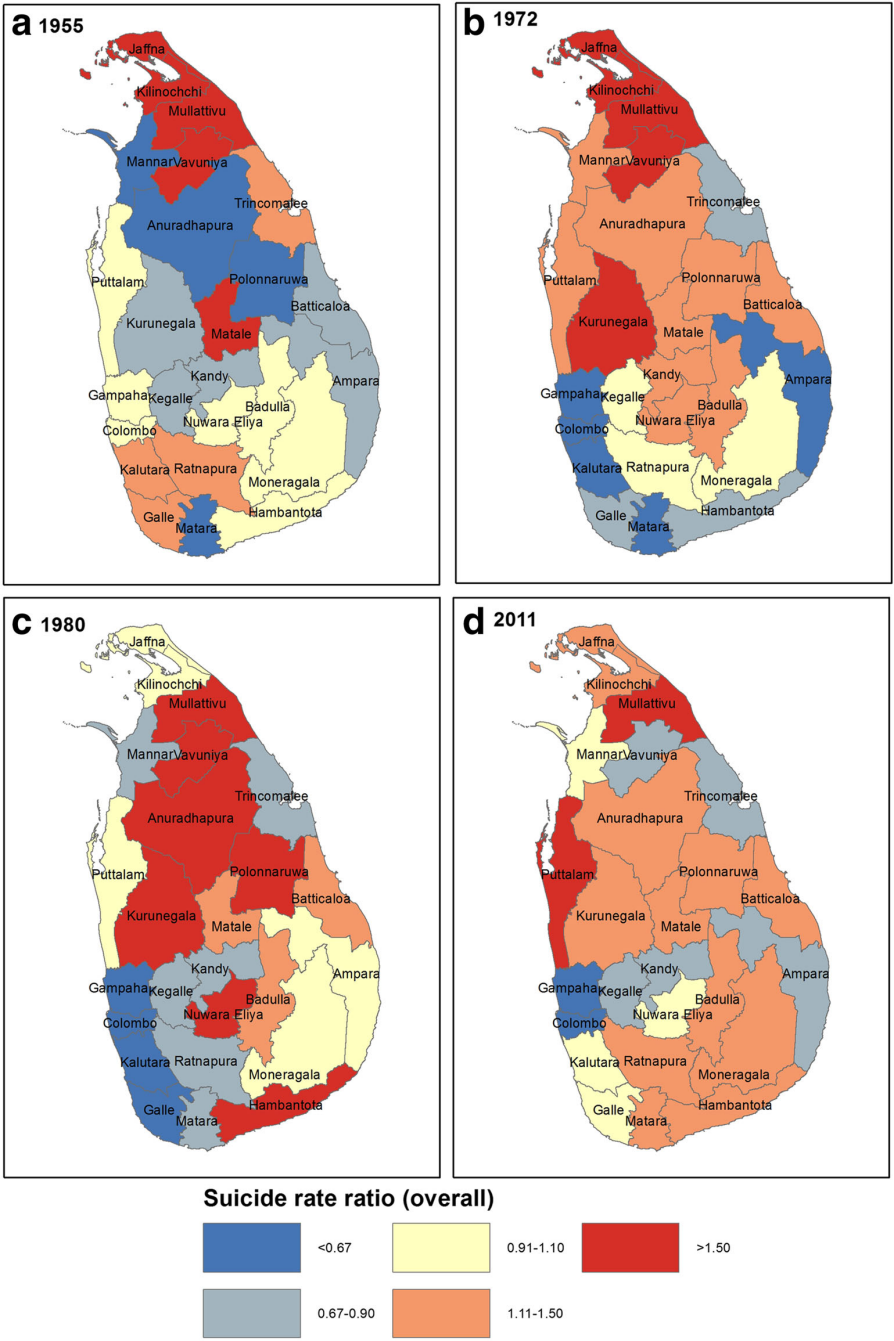
Overall geographic distribution of relative suicide rates in Sri Lanka

Figure 3 illustrates the geographic distribution of relative suicide rates at the four time points. Crude suicide rates by district and maps which illustrate the data disaggregated by sex can be found in Appendix 3 and 4. The lowest rates appear concentrated in and around Sri Lanka’s capital city Colombo throughout the period. The areas with the highest rates are generally in northern and central Sri Lanka, with the exception of 1955.

The district-level trends correspond with those observed nationally, with suicide rates reaching their peak in the majority of districts in 1980 (Appendix 3). In contrast the absolute suicide rates in the urban districts of Colombo and Gampaha remain relatively stable throughout the time period studied.

### Suicide rates stratified by population density

Suicide rates stratified by population density are summarised in Table 2. In all time periods suicide rates are higher in low population density areas. Suicide rates were similar in all three strata in 1955. The biggest difference in suicide rates between population density strata occurred in 1980. The highest relative rate, 3.7, is observed in this year in the most sparsely populated strata, declining to 1.9 in 2011 when overall suicide rates had fallen substantially. The peak in suicide rates in the most populated strata occurs in 1972 in contrast to the peaks in the other two strata, which occur in 1980.

**Table 2:** District-level suicide rates stratified by population density; relative rates estimated using negative binomial models, adjusted models control for district level unemployment rates

Population density (population per Sq. mile)^a^	Suicide rate (per 100,000)				Relative suicide rate (95% confidence intervals)						
	1955	1972	1980	2011	1955*	1972		1980		2011	
					Unadjusted	Unadjusted	Adjusted^b^	Unadjusted	Adjusted^b^	Unadjusted	Adjusted^b^
>2000	6.7	13.3	11.9	11.4	1.0	1.0	1.0	1.0	1.0	1.0	1.0
500–2000	7	22.8	30.4	19.3	1.1 (0.5, 2.1)	1.7 (0.8, 3.5)	1.0 (0.5, 1.7)	2.5 (1.3, 4.7)	2.0 (1.1, 3.4)	1.7 (1.3, 2.3)	1.9 (1.4, 2.5)
<500	7.2	23.9	40.2	22.8	1.1 (0.5, 2.4)	1.9 (0.9, 4.1)	0.7 (0.4, 1.4)	3.7 (2.0, 6.9)	2.2 (1.2, 4.1)	1.9 (1.4, 2.5)	2.1 (1.6, 2.7)

^a^categorised according to 1981 population density; ^b^ Adjusted for unemployment; * district level data for unemployment were unavailable in 1955 so adjusted analysis was not possible

### Census-derived factors

Correlations between suicide rates and various census-derived factors are summarised in Table 3. There was no statistical evidence of an association between ethnicity (%Tamil) or migration, and suicide rates. Population density and unemployment were the only factors for which there was statistical evidence of a correlation with suicide rates at some of the time points investigated. Suicide rates were most strongly correlated with both population density and unemployment in the 1980s.

**Table 3:** Spearman’s rank correlations between census-derived socioeconomic factors and suicide rates over time for Sri Lanka's 25 districts

	Census-derived factors (Spearman’s correlation, r_s_ (*p*-value))			
Years	Population density^a^	Migration^b^	Unemployment^c^	% Tamil
1953–5	−0.10 (0.697)	-	-	0.19 (0.437)
1971–2	−0.40 (0.073)	0.16 (0.487)	−0.55 (0.008)	0.30 (0.171)
1980–1	−0.65 (<0.001)	0.36 (0.084)	−0.62 (0.001)	0.09 (0.665)
2011	−0.49 (0.012)	0.12 (0.578)	−0.08 (0.707)	−0.02 (0.916)

^a^Population/square mile, ^b^% in-migrants, ^c^% aged 10 (1971 & 1981) or 15 (2011) and over and available to work

We note a strong positive correlation between population density and unemployment (0.82 (*p* < 0.001)) i.e. unemployment is low in rural areas. As an additional post-hoc analysis we investigated the association between district level suicide and population density using negative binomial models controlling for district levels of unemployment. Table 2 presents both the unadjusted and adjusted models for the years for which we had data on unemployment. Adjusting for unemployment resulted in an attenuation in the relative rates in the earlier years, but somewhat strengthened associations in 2011.

## Discussion

District-level suicide rates in Sri Lanka have markedly changed over the last 50 years, with evidence that the largest changes are concentrated in the most rural (sparsely populated) areas. The strongest correlation between suicide rates and population density occurred in the 1980s prior to pesticide bans, at a time when access to the most toxic pesticides was at its highest. The stratification of suicide rates by population density elucidates this further; the relative suicide rates in the most rural districts in comparison to the most urban districts before (1955), during (1980) and after (2011) the rise in highly toxic pesticide availability were 1.1 (95% CI 0.5, 2.4), 3.7 (95% CI 2.0, 6.9) and 1.9 (95% CI 1.4, 2.5) respectively. The findings provide some support for the hypothesis that changes in access to pesticides, as indexed by residence in a rural (low population density) area may have contributed to the marked fluctuations in Sri Lanka’s suicide rate [8, 9].

District-level unemployment data were negatively correlated with suicide rates in 1971–2 and 1980–1. This surprising incidental finding is in contrast to a previous study of a single district of Sri Lanka which investigated the geographical patterning of suicide attempts in small communities (median population 1416) [25, 26]. Yet the finding has been noted previously in relation to suicide at district-level [12]. Researchers have speculated about the cause of this negative correlation, suggesting suicides in areas of low unemployment may be the result of people moving from areas of high unemployment in search of work and subsequently experiencing loneliness and isolation [12]. However, districts with low levels of unemployment are also the most sparsely populated (correlation 0.82 (*p* < 0.001)), and this may influence the associations we observed. In models controlling for district levels of unemployment there was still evidence in 1981 and 2011 of a greater rate of suicide in rural areas. The results of these multivariable analyses should, however, be interpreted with caution due to the small sample size (*n* = 25 districts) of this ecological analysis and the strong correlation between area levels of unemployment and population density [27].

This paper also provides support for previous findings that the temporal changes in suicide rates do not correlate with the civil war [8]. The greatest increase in suicide rates in all population density strata occurred in 1972, prior to the war commencing. The peak in suicide rates in the most densely populated strata also occurred in 1972. Furthermore the district-level maps show high suicide rates in a number of central and southern districts that were less affected by the war than areas in the North of the country.

### Strengths and limitations

This study is the first to assess the district-level variation in suicide rates in Sri Lanka over the time period during which there were marked changes in suicide rates, and to correlate these changes with secular variations in important social factors. Our use of maps enables the changing spatial distribution of suicide to be easily visualised.

The findings in this study should however be considered in the context of the following limitations. Firstly, in our analysis we were unable to obtain a direct measure of access to pesticides. We therefore used population density as a proxy indicator for rurality and thereby pesticide access. Population density is a frequently used measure of rurality [19, 20] and pesticide poisonings have been shown to occur more commonly in rural areas [28, 29]. It has been argued that population density may not always be the most appropriate measure of rurality [30]. This is especially the case in large districts where the majority of people reside in an urban centre; when population density is used to classify the population in these districts, they would be incorrectly categorised as rural residents. Nevertheless, based on our knowledge of Sri Lanka, a review of the way each district was categorised in our study reassures us that this was not the case in our study. To explore the use of population density as a proxy indicator further, we investigated the correlation between population density and percentage of agricultural workers per district in 2011. The high correlation provided additional support for this approach (−0.58; *p* = 0.025) [15].

Secondly, method-specific district-level suicide mortality data were unavailable. National data however suggest since their introduction in Sri Lanka, pesticides have been the most common method of suicide [8] for example accounting for 55% of suicides in 2005 [31]. Furthermore the percentage of pesticide suicides varies over time and previous analyses have indicated that most of the temporal trends in suicide in Sri Lanka are driven by changes in pesticide self-poisoning [8, 9].

Thirdly, we were unable to investigate the relationship between mental illness and suicide rates in each time period, as district-level data on mental illness were only available for 2012 [32]. Compared to the West, mental illness in Asia appears to contribute to a relatively smaller proportion of suicides [21, 33]. Data from Sri Lanka suggest that only 48% of suicide deaths [34] and 52% of self-poisoning attempts screened positive for depression [35].

Fourthly, there may be area variations in the quality of the recording of suicide due to differences in recording processes and stigma. These errors however are most likely to lead to under-estimation of the strength of associations. Fifthly, there were a number of changes in definitions and administrative boundaries for data collection over time. As described earlier, we assumed suicide rates for new districts were the same as for the districts from which they were formed. With the exception of one district, the 2011 suicide rates were generally similar between the new districts and the old ones from which they were formed (see Appendix 2). Ethnicity is an example of a potential risk factor for which there have been changes in definition over time. In 1953, Tamil ethnicity was recorded as a single category, whereas in later censuses Tamils were divided into Sri Lankans and Indians. It is also possible that there are more subtle changes in data collection that are not apparent in the census reports. It may therefore be more appropriate to interpret the risk factor data as stand-alone years, rather than over time. Finally, it is important to emphasise that these results may be subject to ecological fallacy, therefore causal inferences are limited.

### Public health implications

This research lends support to previous studies, which have suggested that suicide rates are influenced by access to pesticides in Sri Lanka. The findings can to some extent be extrapolated to other populous middle-income countries in Asia, most notably India and China. These two countries are the biggest manufacturers of pesticides globally [36] and have a large agricultural sector, heavy pesticide use and high levels of pesticide self-poisoning [2, 37, 38]. Yet neither country has enforced as many bans on the most toxic pesticides as Sri Lanka and neither country has achieved the same level of reduction in suicide rates during the time period studied [39, 40]. Since 2006 a downturn in the incidence of suicides in China has been observed. However this is thought to be largely due to economic prosperity and population shifts from rural to urban areas [29]. Consequently the findings of this study indicate that the World Health Organisation’s call to restrict the most toxic pesticides globally, could also have a major impact on suicide levels elsewhere in Asia [41].

## Conclusion

Bearing in mind the limitations of this study, district-level variation in suicide rates provides some support for the hypothesis that the fall in suicide rates was driven by reductions in access to lethal pesticides.

## Figures and Tables

**Fig. 4:**
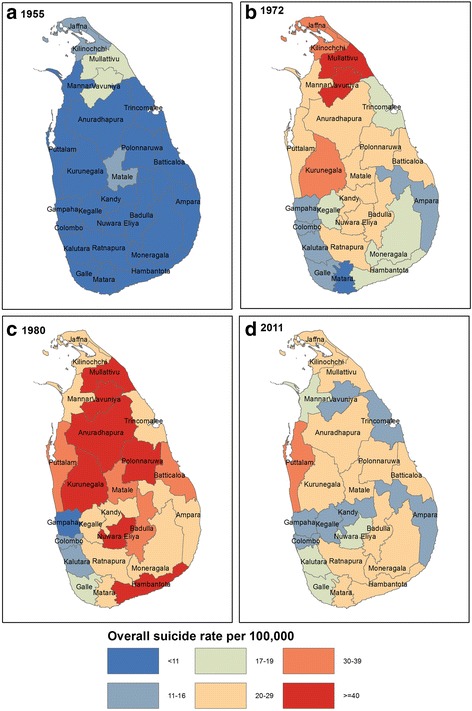
Geographic distribution of overall absolute suicide rates over four time points

**Fig. 5:**
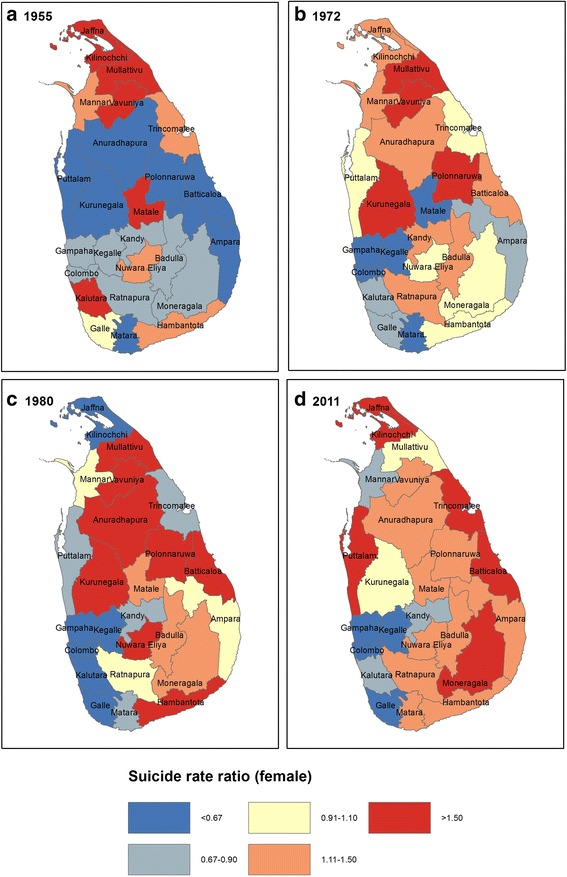
Geographic distribution of relative suicide rates amongst women over four time points

**Fig. 6:**
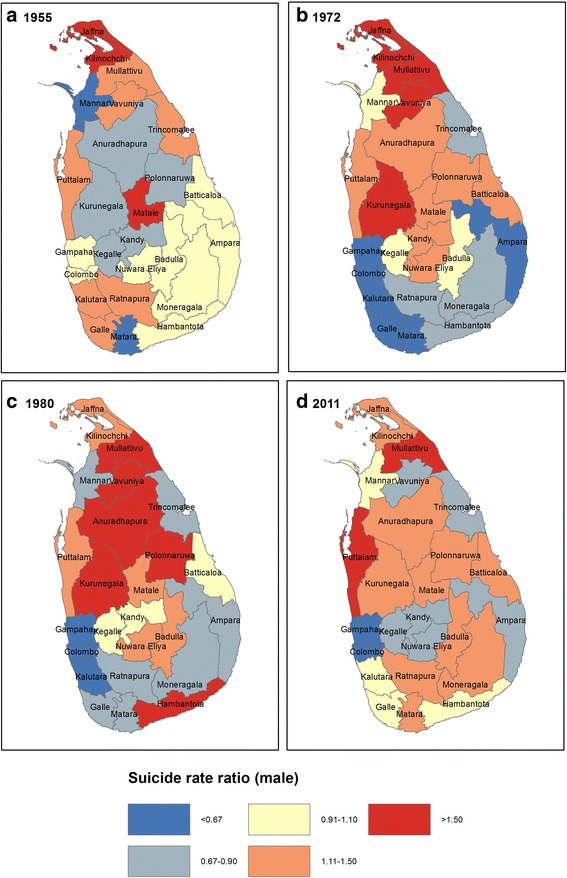
Geographic distribution of relative suicide rates amongst men over four time points

## Appendix 1: Police divisions and relevant districts

**ᅟ:** ᅟ

	Police Division	District
1	Ampara	Ampara
2	Anuradhpura	Anuradhpura
3	Badulla	Badulla
4	Bandarawela	Badulla
5	Batticaloa	Batticaloa
6	Chilaw	Puttlam
7	Colombo North	Colombo
8	Colombo South	Colombo
9	Colombo Central	Colombo
10	Elpitiya	Galle
11	Galle	Galle
12	Gampaha	Gampaha
13	Gampola	Kandy
14	Hatton	Nuwara Eliya
15	Jaffna	Jaffna
16	Kalutara	Kalutara
17	Kandy	Kandy
18	Kantale	Trincomalee
19	Kegalle	Kegalle
20	Kelaniya	Gampaha
21	Kuliyapitya	Kurunegala
22	Kurunegala	Kurunegala
23	Matale	Matale
24	Matara	Matara
25	Monaragala	Monaragala
26	Mount Lavinia	Colombo
27	Negombo	Gampaha
28	Nikaweratiya	Kurunegala
29	Nugegoda	Colombo
30	Nuwara Eliya	Nuwara Eliya
31	Pandura	Kalutara
32	Polonnaruwa	Polonnaruwa
33	Ratnapura	Ratnapura
34	Tangalle	Hambantota
35	Trincomalee	Trincomalee
36	Vavuniya	Vavuniya
37	lativu	Mullativu
38	Mannar	Mannar
39	Puttlam	Puttlam
40	Kankasanthure	Jaffna
41	Mankulam	Mullativu
42	Kilinochchi	Kilinochchi
43	Seetawakapura	Colombo

## Appendix 2: Comparison of suicide rates in 2011 between new districts and the original districts from which they were formed

**ᅟ:** ᅟ

New district	Suicide rate (95% confidence intervals)	Original district	Suicide rate (95% confidence intervals)
Ampara	15.4 (12.5–18.7)	Batticaloa	21.6 (20.0–28.3)
Gampaha	11.2 (9.9–12.7)	Colombo	11.5 (10.2–13.0)
Mullativu	28.2 (18.4–41.3)	Jaffna	24.5 (20.6–28.9)
Kilinochchi	27.3 (18.6–38.8)	Jaffna	24.5 (20.6–28.9)

## Appendix 3: District-level absolute suicide rates

**ᅟ:** ᅟ

District	Crude Suicide rate per 100,000			
	1955	1972	1980	2011
Ampara	5.7	12.0	26.5	15.4
Anuradhapura	3.8	24.4	48.4	24.2
Badulla	6.7	24.2	37.8	21.6
Batticaloa	5.7	25.4	39.9	23.7
Colombo	6.7	13.3	13.7	11.5
Galle	7.8	14.1	18.6	17.1
Gampaha	6.7	13.3	9.8	11.2
Hambantota	7.5	18.0	53.6	21.3
Jaffna	13.1	32.9	27.4	24.5
Kalutara	9.8	12.9	11.7	17.1
Kandy	5.1	23.9	25.8	15.6
Kegalle	4.9	19.9	23.0	12.5
Kilinochchi	13.1	32.9	27.4	27.3
Kurunegala	4.8	36.7	58.9	24.3
Mannar	4.3	24.1	25.1	17.1
Matale	11.0	29.2	36.2	23.3
Matara	2.7	9.8	23.0	21.0
Moneragala	6.7	19.0	28.4	25.7
Mullaitivu	17.9	63.9	89.3	28.2
Nuwara	7.0	24.3	44.3	17.0
Polonnaruwa	3.8	28.3	50.2	24.1
Puttalam	7.3	25.0	30.7	30.6
Ratnapura	8.9	21.3	24.0	22.6
Trincomalee	8.7	18.3	21.6	16.3
Vavuniya	17.9	63.9	84.1	15.7
